# Reflections on Modeling Poliovirus Transmission and the Polio Eradication Endgame

**DOI:** 10.1111/risa.13484

**Published:** 2020-04-27

**Authors:** Kimberly M. Thompson, Dominika A. Kalkowska

**Affiliations:** ^1^ Kid Risk, Inc. Orlando FL USA

**Keywords:** dynamic modeling, eradication, oral poliovirus vaccine, polio

## Abstract

The Global Polio Eradication Initiative (GPEI) partners engaged modelers during the past nearly 20 years to support strategy and policy discussions and decisions, and to provide estimates of the risks, costs, and benefits of different options for managing the polio endgame. Limited efforts to date provided insights related to the validation of the models used for GPEI strategy and policy decisions. However, modeling results only influenced decisions in some cases, with other factors carrying more weight in many key decisions. In addition, the results from multiple modeling groups do not always agree, which supports selection of some strategies and/or policies counter to the recommendations from some modelers but not others. This analysis reflects on our modeling, and summarizes our premises and recommendations, the outcomes of these recommendations, and the implications of key limitations of models with respect to polio endgame strategy. We briefly review the current state of the GPEI given epidemiological experience as of early 2020, which includes failure of the GPEI to deliver on the objectives of its 2013–2018 strategic plan despite full financial support. Looking ahead, we provide context for why the GPEI strategy of global oral poliovirus vaccine (OPV) cessation to end all cases of poliomyelitis looks infeasible given the current state of the GPEI and the failure to successfully stop all transmission of serotype 2 live polioviruses within four years of the April–May 2016 coordinated cessation of serotype 2 OPV use in routine immunization.

## 1 INTRODUCTION

Prospective modeling continues to play a critical role in analytic‐deliberative processes by supporting the evaluation of strategies and decisions for managing risks in complex systems. However, modelers rarely perform validation exercises to explore whether the estimates made prior to actions and events occurring proved correct, and to identify and learn from any significant errors in the case of poor estimates (Thompson, Segui‐Gomez, & Graham, 2002). The importance of using real‐world data after implementation of decisions to assess actual costs and benefits emerges as one key lesson of validation exercises (Hahn & Tetlock, 2008). Comparing what actually happened to what was expected may reveal poor assumptions about model inputs and/or the system. However, despite uncertainties in the model inputs, particularly related to future events, validation efforts can evaluate the robustness of the decisions and overall path taken and assess whether the insights and conclusions of the analysis proved valid at a high level (Thompson et al., 2002).

**Fig 1:**
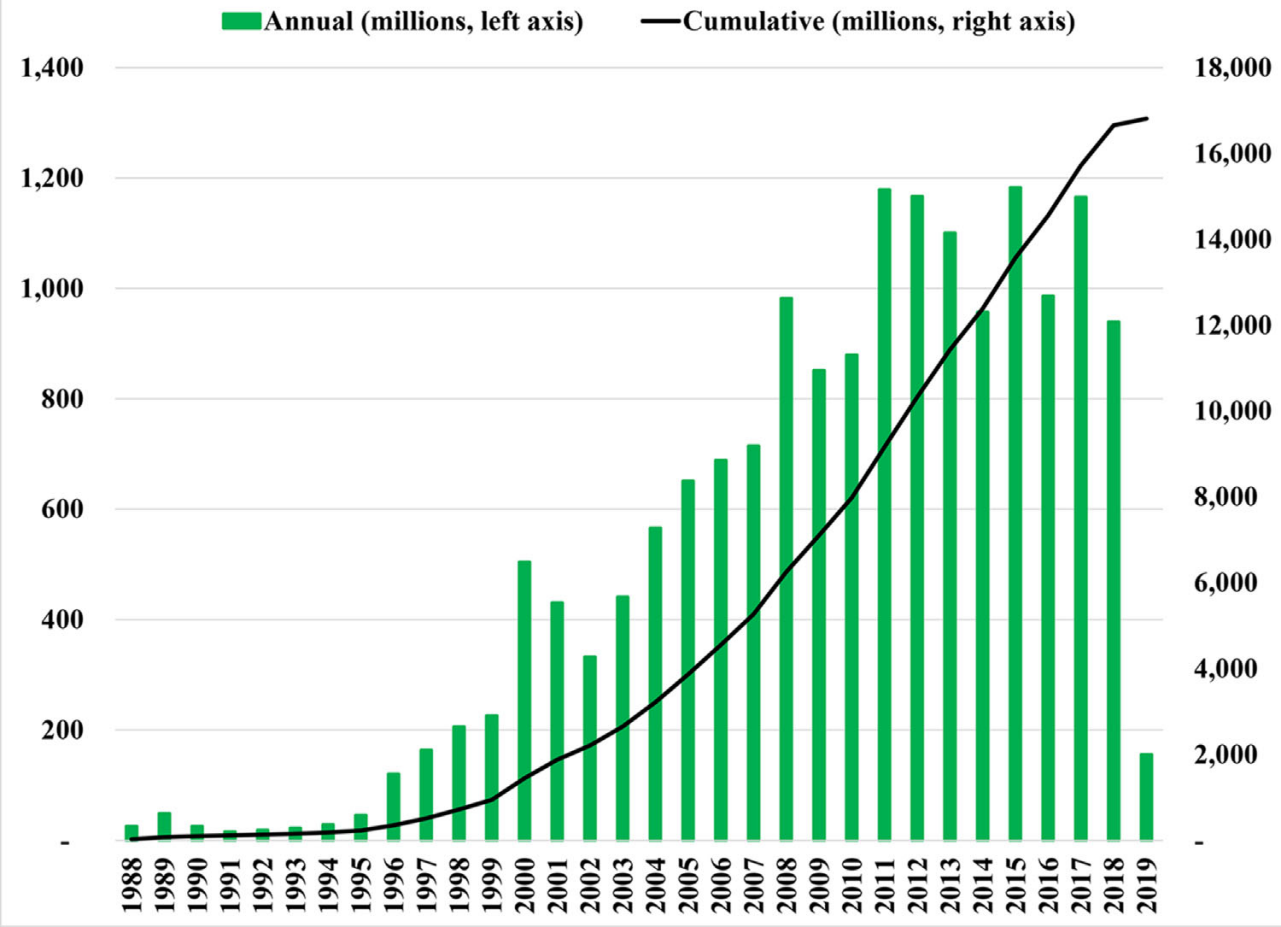
Historical contributions to the GPEI by year 1988–2019* (World Health Organization Global Polio Eradication Initiative, 2020 **)**. *2019 reporting incomplete.

Following a 1988 World Health Assembly resolution to eradicate polio by the year 2000 (World Health Assembly, 1988), the Global Polio Eradication Initiative (GPEI) began its efforts to stop and prevent the transmission of all three serotypes of wild polioviruses (WPVs) and ultimately end all cases of poliomyelitis (hereafter polio). Countries and the GPEI achieved numerous successes leading to a significant decline in polio cases. Notably, the GPEI developed strategies and raised financial resources for their implementation to help support countries with national immunization programs that failed to stop poliovirus transmission on their own before 1989 (Duintjer Tebbens et al., 2011). Fig. 1 summarizes contributions from donors for the GPEI between 1988 and 2019 (2019 reporting not complete) (World Health Organization Global Polio Eradication Initiative, 2020). The primary strategy to increase immunization coverage depends on conducting supplemental immunization activities (SIAs) using oral poliovirus vaccine (OPV), which increase immunization coverage beyond the levels achieved by national age‐schedule‐based routine immunization (RI). SIAs typically include large, planned and preventive campaigns (pSIAs) that reach large numbers of children within a specific age range independent of prior immunization, but SIAs can also include reactive, outbreak response campaigns (oSIAs). The GPEI supports both types of SIAs. Eradication of polioviruses implies permanent prevention of transmission, which means that cases (and the need to conduct oSIAs) represent a failure with respect to achieving the ultimate goal. Thus, countries and the GPEI appropriately focus primarily on pSIAs using OPV to prevent transmission in areas with low RI coverage.

Despite receiving the full financial support from donors and broad global health community support for its 2013–2018 GPEI Strategic Plan (World Health Organization Global Polio Eradication Initiative, 2013), which the GPEI extended to cover 2013–2019 following a midterm review (World Health Organization Global Polio Eradication Initiative, 2015), polio eradication is not done. The goals of achieving eradication of all WPVs, ending all cases of poliomyelitis, and delivering on the promises of the 2013–2019 GPEI Strategic Plan remain elusive. Section 2 provides an overview of the current status of the GPEI and polio epidemiology as of the beginning of 2020, but prior to the Coronavirus Disease 2019 (COVID‐19) pandemic.

Over the past nearly 20 years, the GPEI partners engaged modelers to support strategy and policy discussions and decisions by providing estimates of the risks, costs, and benefits of different options for managing the polio endgame. Models may differ in numerous ways, including their framing, assumptions, and intended uses. We developed integrated models that combined risk and decision analytic, economic, and system dynamic techniques according to the premises discussed in Section 3. Section 4 summarizes some of the key strategies and policies our models recommended over the last 19 years. Section 5 compares our recommendations to the actual GPEI path (i.e., GPEI and country decisions and what occurred), and highlights that our modeling results only influenced the GPEI and national decisions in some cases, with other factors carrying more weight in many key decisions. Section 6 summarizes the lessons we learned from the cessation of serotype 2‐containing OPV (OPV2) reflecting on four years of evidence and implications for OPV cessation as a strategy. Section 7 discusses the implications of using models to support the GPEI with a perspective on the road ahead.

## 2 CURRENT STATUS OF THE GPEI AND POLIO EPIDEMIOLOGY

As of 2020, the GPEI still seeks to achieve its mission of ending all cases of poliomyelitis, which requires stopping and preventing the transmission of all three serotypes (1, 2, and 3) of WPV (i.e., WPV1, WPV2, and WPV3) (World Health Assembly, 1988). Countries and the GPEI successfully stopped all global transmission of WPV2 before 2000, and the GPEI Global Certification Commission (GCC) certified WPV2 eradication in September 2015 (World Health Organization, 2016a). Nigeria reported the last paralytic case caused by serotype 3 WPV in November 2012 (World Health Organization, 2016c), and the GCC certified WPV3 eradication in October 2019 (World Health Organization, 2019). Finally, WPV1 transmission continues to date in Pakistan and Afghanistan, with the disturbing trend of 22, 33, and 175 reported paralytic cases caused by WPV1 in 2017, 2018, and 2019, respectively (2019 data as of March 27, 2020) (World Health Organization, 2020).

Preventing all cases of poliomyelitis (World Health Assembly, 1988) will depend on successfully ending all use of OPV after certification of WPV eradication, because OPV can cause rare cases of vaccine‐associated paralytic polio (VAPP) in fully susceptible OPV vaccine recipients and their close contacts, and can cause paralysis in fully susceptible individuals who become infected by transmission of OPV‐related viruses known as vaccine‐derived polioviruses (VDPVs). Live polioviruses (LPVs, i.e., OPV, OPV‐related viruses, and VDPVs) vary in their ability to transmit and cause paralysis and by serotype (see Table I). Following the certification of WPV2 eradication, the GPEI globally coordinated the cessation of all use of OPV2 in late April and early May 2016, except for emergency use of monovalent serotype 2 OPV (mOPV2) to respond to serotype 2 outbreaks (World Health Organization, 2016b). While most OPV‐using countries successfully stopped OPV2 use and experienced die out of transmission of all serotype 2 LPVs (Thompson & Duintjer Tebbens, 2017b), unfortunately OPV2 cessation did not go smoothly everywhere (Duintjer Tebbens & Thompson, 2018). Compared to the 33 reported cases caused by WPV1 in 2018, transmission of serotype 2 circulating vaccine‐derived polioviruses (cVDPV2s) caused 71 reported cases in five countries in 2018 and 353 cases in 16 countries in 2019 (data as of March 27, 2020) (World Health Organization, 2020). Responding to cVDPV2 transmission continues to lead to the use of mOPV2, which raises questions about whether the coordinated global OPV2 cessation of mid‐2016 can ultimately succeed and what this means for OPV cessation as a polio endgame strategy (Duintjer Tebbens & Thompson, 2018; Thompson & Kalkowska, 2019).

**Table I:** Relative Serotype‐Specific Model Inputs for Transmissibility (*R*_0_) and Paralysis‐to‐Infection Ratios (PIRs) by Serotype (Duintjer Tebbens et al., 2013)

Model Input	Serotype 1	Serotype 2	Serotype 3
Relative basic reproduction number (*R*_0_) for WPV or fully reverted VDPV (relative to serotype 1)^a^	1 (reference)	0.9	0.75
Relative *R*_0_ of OPV parent strain to WPV or VDPV (relative to row above for same serotype)^a^	0.37	0.55	0.25
Average paralysis‐to‐infection ratio for fully susceptible individuals			
WPV OPV	1/2000.26 × 10^−6^	1/2,0001.2 × 10^−6^	1/1,0001.2 × 10^−8^

OPV = oral poliovirus vaccine; VDPV = vaccine‐derived poliovirus; WPV = wild poliovirus.

^a^ Thus, for a country with an assumed average *R*_0_ = 10 for WPV1, this implies *R*_0_ = 9 for WPV2, *R*_0_ = 7.5 for WPV3, *R*_0_ = 3.7 for OPV1, *R*_0_ = 4.95 for OPV2, and *R*_0_ = 1.875 for OPV3.

Recently, the GPEI issued a new Strategic Plan for 2019–2023 (World Health Organization Global Polio Eradication Initiative, 2019) and the GPEI now needs to develop financial support for its new multiyear plan. The new plan contrasts sharply with earlier GPEI expectations of transitioning GPEI assets and responsibilities to countries as of the end of 2018 (or 2019) (World Health Organization Global Polio Eradication Initiative, 2019). Notably, the GPEI had already begun some of its transition activities by decreasing resources to some countries and activities that it previously supported (World Health Organization, 2017b).

The GPEI continues to seek support from modelers to make the case for its current polio endgame plans, because modeling played a role in evaluating and guiding GPEI strategies and policies to date. Since multiple polio modeling groups support the GPEI with differing approaches, the next section provides an overview of the premises that guide our modeling and the following section highlights some of our key recommendations.

## 3 KEY PREMISES OF OUR MODELING

Identifying the premises used by modeling can provide context that should help identify areas of disagreement due to different premises held by others. LPV infections in some fully susceptible individuals can cause paralysis (i.e., poliomyelitis) that can leave individuals with life‐long severe disability and in rare cases cause death (Sutter, Kew, Cochi, & Aylward, 2017). In addition, dealing with polio implies significant costs for affected individuals and their caregivers (i.e., disability and productivity losses), health systems (i.e., treatment costs), and societies (i.e., fear, stigma, opportunity costs, and productivity losses) (Bart, Foulds, & Patriarca, 1996; Duintjer Tebbens, Pallansch, Wassalik, Cochi, & Thompson, 2015a; Musgrove, 1988).

Premise 1 As long as LPVs circulate, they will cause cases of paralysis that will lead to health and financial costs.

Corollary 1.1 WPV eradication offers equity by ensuring permanent prevention of exposure to potential WPV transmission for everyone.

Collollary 1.2 National choices about poliovirus vaccines lead to different health and financial costs.

Countries can avoid WPV‐related health costs by investing in polio immunization (Bart et al., 1996; Musgrove, 1988; Thompson & Duintjer Tebbens, 2006, 2007) so long as LPVs transmit. Important differences exist in the risks, costs, and benefits of OPV and inactivated poliovirus vaccine (IPV), including the different nature of the immunological protection that they induce (Duintjer Tebbens & Thompson, 2017d; Sutter et al., 2017; Vidor, 2017) and between the serotypes (Duintjer Tebbens et al., 2015a; Thompson & Duintjer Tebbens, 2017b). Nearly all countries that eliminated WPV transmission prior to the creation of the GPEI needed to use OPV to achieve sufficient population immunity to stop transmission (Sutter et al., 2017), although a small number of countries with very high functioning health systems, good hygiene, and temperate climates apparently stopped WPV transmission using only IPV (e.g., The Netherlands, Iceland) (Vidor, 2017). After successful national elimination of transmission of WPVs and in the context of low (but not zero) risks of WPV importation from other countries, some relatively high‐income countries with good health systems chose to switch to RI schedules that used IPV only or IPV then OPV (i.e., IPV/OPV sequential schedules) to avoid rare cases of VAPP (Thompson & Duintjer Tebbens, 2006; Vidor, 2017), despite the unfavorable cost‐effectiveness of using IPV (Miller, Sutter, Strebel, & Hadler, 1996). One country that switched to an IPV‐only schedule (i.e., Israel) needed to restart the use of OPV in the form of an IPV/OPV schedule following evidence of transmission of imported WPV1 despite its very high IPV‐only coverage (Anis et al., 2013; Kalkowska, Duintjer Tebbens, Grotto, et al., 2015).

Premise 2 OPV is the vaccine of choice for stopping WPV transmission, because it induces mucosal immunity, spreads secondarily, and is cheaper and easier to deliver than IPV. Countries that cannot stop or prevent transmission with OPV will not be able to stop or prevent transmission with IPV.

Corollary 2.1 Successful polio eradication depends on sufficient quantities of OPV and its effective delivery, which depends on a commitment to use OPV, despite its risks.

Corollary 2.2 IPV plays little role in maintaining or sustaining population immunity to transmission, although it protects individuals who only receive IPV from paralysis if they become infected with a LPV.

Corollary 2.3 National immunization programs do not and will not all reach 100% coverage, and consequently, equity in delivery of vaccine will not occur in practice and some individuals will remain susceptible.

In countries relevant to polio eradication (i.e., those who did not achieve national elimination prior to establishment of the GPEI), review of data and experience shows that RI alone did not achieve and maintain sufficiently high immunization coverage to eliminate and prevent poliovirus transmission, which led to the need for SIAs (Hull, Ward, Hull, Milstien, & de Quadros, 1994). Even with strong RI programs in the WHO Region of the Americas, the Pan American Health Organization (PAHO) strategy to increase immunity in the population included performing pSIAs, which it called “national immunization days” and oSIAs with OPV (Olive, Risi, & de Quadros, 1997).

Premise 3 Countries that did not successfully eliminate WPV transmission with their RI programs and that do not achieve high coverage need to conduct SIAs with OPV.

Corollary 3.1 In countries with failing RI programs, achieving polio eradication requires temporarily establishing (and paying for) the infrastructure required to conduct SIAs to achieve high coverage (i.e., the GPEI cannot “strengthen” health systems that do not exist, and in order to succeed, the GPEI needs to work with countries to create functional and accountable teams that perform the polio immunization tasks necessary to achieve and maintain national elimination until successful global eradication).

Corollary 3.2 GPEI investments in infrastructure may support other activities (i.e., provide external benefits), for which the GPEI can claim some credit (proportional to its support), but the GPEI should support only those investments required to achieve polio eradication.

Corollary 3.3 After successful polio eradication, temporary investments made to support GPEI infrastructure could/can stop and countries will achieve the RI coverage that their national budgets and systems support.

Corollary 3.4 After successful polio eradication, the dividends from the eradication effort would include savings associated with avoided treatment costs, productivity losses, and fear associated with polio outbreaks and cases, and savings associated with no longer needing to pay for OPV in RI and in SIAs (i.e., countries that want to continue with any polio immunization due to their concerns about bioterrorism or other risks could continue with expensive IPV in RI, but other countries could stop polio vaccination altogether and save all associated costs).

Eradication efforts require significant investment of resources, which must come from internal (i.e., national) and external (i.e., donor) funding sources. Donors expect good returns on their investments, in this case, permanently ending the scourge of polio in exchange for funds provided.

Premise 4 The GPEI seeks to achieve polio eradication as quickly and as cost‐effectively as possible.

Corollary 4.1 GPEI donors care about how their funds are used, and want them to be used to cost‐effectively achieve polio eradication.

Corollary 4.2 The GPEI is not a funding mechanism for external donors to pay to build health systems in countries that have chosen not to create or maintain their own systems, although GPEI funds that create infrastructure to deliver polio vaccines may improve existing health systems and/or create new capacities that countries could later incorporate into their national health systems.

Eradication is all or nothing, and the potential timing and success depends on overcoming the weakest links (Barrett, 2009). Review of experience showed the failure to vaccinate (i.e., not vaccine failure) as the cause of outbreaks (Patriarca, Sutter, & Oostvogel, 1997).

Premise 5 Achieving WPV eradication requires contemporaneously attaining sufficiently high population immunity to transmission in all countries for all three serotypes to stop all WPV transmission.

Corollary 5.1 After a country successfully disrupts its indigenous WPV transmission, it needs to continue to maintain high population immunity to transmission to prevent any WPV exported by other countries into its borders from reestablishing transmission and causing outbreaks.

Corollary 5.2 The faster that the GPEI can help countries ramp up their polio immunization and surveillance infrastructure to achieve and maintain sufficiently high population immunity to transmission, the more quickly eradication can occur.

Corollary 5.3 Polio eradication infrastructure needs to stay in place until successful eradication.

When OPV‐using countries achieve high coverage with OPV, cases of VAPP can exceed cases of WPV (Vidor, 2017), which can raise issues related to the acceptability of OPV and potentially lead to lower coverage. When OPV‐using countries do not achieve and maintain high coverage, the transmission of OPV‐related viruses can lead to cVDPV outbreaks (Kew et al., 2002).

Premise 6 OPV‐using countries should only use OPV that contains all of the serotypes of OPV globally in use (i.e., use only tOPV prior to cessation of any OPV serotype, use only bivalent OPV [bOPV] after OPV2 cessation but prior to globally‐coordinated cessation of serotypes 1 and 3 OPV) in order to avoid creating serotype‐specific immunity gaps.

Corollary 6.1 If OPV use leads to the ongoing transmission of OPV‐related viruses that cause cVDPVs, then population immunity to transmission is too low and intensification of OPV immunization needs to occur to increase coverage.

Corollary 6.2 After ending indigenous WPV transmission, countries need to maintain high population immunity to transmission to prevent cVDPVs, which will mean continuing to conduct SIAs in countries with insufficient RI coverage (same action as Corollary 5.1, but for managing cVDPV risks instead of WPV importation risks).

Corollary 6.3 Polio eradication infrastructure needs to stay in place until successful cessation of all OPV use.

Stopping outbreaks typically requires oSIAs with OPV, and the characteristics of these oSIAs (i.e., timing, quality, and scope) will determine their ability to raise population immunity to transmission sufficiently to stop transmission of the outbreak virus (Duintjer Tebbens, Pallansch, Wassilak, Cochi, & Thompson, 2016; Thompson, Duintjer Tebbens, & Pallansch, 2006).

Premise 7 Countries and the GPEI should seek to perform high‐quality, timely oSIAs with OPV of sufficient scope to respond to outbreaks.

Corollary 7.1 Compared to OPV, IPV does not provide the same immunological protection and IPV is more difficult to deliver in outbreak settings, and consequently, the GPEI and countries should use OPV for outbreak response if available.

Corollary 7.2 So long as it might be needed, the GPEI and countries should ensure sufficient availability of OPV for outbreak response.

The use of OPV complicates the polio endgame, because even though high coverage with OPV is required to achieve eradication, OPV causes VAPP and VDPVs (Dowdle, de Gourville, Kew, Pallansch, & Wood, 2003).

Premise 8 Ending all cases of poliomyelitis depends on stopping all use of OPV and successfully containing all LPVs.

Corollary 8.1 Globally coordinated OPV cessation of each serotype in all OPV‐using countries will reduce the risks of countries importing homotypic OPV from other countries.

Recognizing polio eradication as a global major project, planning for the polio endgame should include the creation of options to help manage post‐WPV eradication risks.

Premise 9 After successful WPV eradication, achieving successful OPV cessation and maintenance of a polio‐free world will require some additional resources and planning.

Corollary 9.1 After WPV‐eradication and OPV cessation, population immunity to transmission will decrease and reintroduction of LPVs will threaten eradication.

Corollary 9.2 IPV use will not significantly decrease the chances of transmission but will provide some expensive insurance in the form of somewhat reduced numbers of paralyzed children if LPV reintroduction occurs, with the extent of protection depending on the RI coverage for IPV.

Corollary 9.3 Preventing reintroduction of LPVs will also require effective LPV containment and management of potential risks of immunodeficiency‐associated VDPVs (iVDPVs).

## 4 KEY RECOMMENDATIONS OF OUR MODELING

Our modeling suggests that the GPEI should pursue the following actions:

1. Develop and maintain high‐quality poliovirus surveillance to enable rapid identification of any LPV transmission and its potential or likely source (i.e., “actively look”).
2. Identify countries (or subpopulations within countries) with insufficient population immunity to transmission and work with them to create accountable infrastructure to conduct OPV pSIAs to increase their population immunity to transmission using OPV containing all serotypes in current use (i.e., “go big with OPV”)
3. Maintain the infrastructure required to sustain performance and to achieve high coverage with OPV prior to its cessation (i.e., “stay big with OPV”).
4. Ensure accountability at all levels to performance goals (i.e., “don't tolerate bad performance”).
5. Plan for the polio endgame and manage financial risks by developing multiyear plans and budgets that will ensure sufficient resources to maintain population immunity to transmission using OPV (i.e., “treat polio eradication as a global major project”).
6. Recognize that the GPEI's success depends on excellent leadership and management (i.e., “ensure good leadership”).
7. Ensure sufficient availability of OPV by working with manufacturers and countries to support planned activities and create stockpiles (i.e., “ensure that you have the vaccine resources you need and recognize vaccine manufacturers as partners”).
8. Prior to OPV cessation, in areas with low OPV coverage, intensify population immunity to transmission in subpopulations with low population immunity by conducting OPV pSIAs prior to OPV cessation (i.e., “prevention of cVDPVs is better than chasing them reactively”).
9. Respond to any outbreaks by rapidly conducting high‐quality oSIAs with sufficient numbers of rounds to prevent breakthrough transmission, and if breakthrough transmission occurs, conduct additional rounds (i.e., “respond quickly and strong”).
10. Recognize IPV as a risk management option for countries that want it after OPV cessation, but NOT a requirement for all countries, and help to ensure tiered pricing for IPV such that any countries that want it can afford IPV should they choose to pay for it (i.e., “IPV is an option, not a requirement or a right, and it will cost real resources”).
11. If WPV1 eradication is not feasible, plan for many countries that currently use OPV to pursue control with tOPV, since these countries will not likely wish to continue to pay for both OPV and relatively more expensive IPV, donor support for IPV will likely disappear at some point, IPV will not stop WPV transmission, and OPV will be necessary for oSIAs, and some countries will prefer OPV (i.e., “ensure access and availability of polio vaccines that countries will prefer for long‐term control, if needed”)
12. Develop plans to facilitate the smoothest possible transition back to a world that uses tOPV in the event that a polio‐free world fails (i.e., “develop contingencies”).

We made numerous recommendations to the GPEI consistent with these actions, which we group into themes.

### 4.1 Poliovirus Surveillance

Our modeling supported investments in poliovirus surveillance by estimating the global polio laboratory network costs (de Gourville, Sangrujee, Duintjer Tebbens, Pallansch, & Thompson, 2006; Duintjer Tebbens, Diop, Pallansch, Oberste, & Thompson, 2019), reviewing the literature related to poliovirus environmental surveillance (Duintjer Tebbens, Zimmermann, Pallansch, & Thompson, 2017), and showing the value of poliovirus surveillance with respect to managing risks (de Gourville et al., 2006; Duintjer Tebbens et al., 2016; Thompson et al., 2006). We also demonstrated the role of poliovirus surveillance in increasing the confidence about no undetected circulation as a function of time since the last detected event (i.e., reported acute flaccid paralysis [AFP] case or detection from an environmental sample) (Duintjer Tebbens, Kalkowska, & Thompson, 2019; Kalkowska, Duintjer Tebbens, Grotto, et al., 2015; Kalkowska, Duintjer Tebbens, Pallansch, et al., 2015; Kalkowska, Duintjer Tebbens, & Thompson, 2012; Kalkowska, Duintjer Tebbens, Pallansch, & Thompson, 2019). We recommended that the GPEI and countries maintain very high‐quality AFP surveillance throughout WPV eradication and OPV cessation and we assumed that they would do so in our modeling (Duintjer Tebbens et al., 2015a; Thompson et al., 2008). Given our early recognition of the potential for LPV transmission to occur in some populations with high IPV coverage based on our U.S. modeling (Thompson et al., 2012), the discovery of transmission of imported WPV1 in Israel by its extensive environmental surveillance system demonstrated that prospective modeling could anticipate potential events prior to their observation and led us to recommend OPV use to stop and prevent WPV1 transmission in Israel (Anis et al., 2013; Kalkowska, Duintjer Tebbens, Grotto, et al., 2015). With the GPEI investing significantly in expanding poliovirus environmental surveillance, we recommended better characterization of environmental surveillance systems given the limited role of environmental surveillance to date (Duintjer Tebbens et al., 2017), and challenges associated with understanding the value of environmental surveillance in Pakistan and Afghanistan (Duintjer Tebbens, Pallansch, et al., 2019; Kalkowska & Thompson, 2019).

### 4.2 Population Immunity to Transmission

Our modeling in support of accelerating eradication repeatedly recommended that the GPEI and countries achieve and maintain sufficiently high population immunity to transmission for all serotypes. In 2007, we recommended the GPEI and countries not waver in their commitments to polio eradication (Thompson & Duintjer Tebbens, 2007) and recognized the need to ensure sufficient financial resources to keep immunization intensity (i.e., population immunity to transmission) high until successful WPV eradication. As delays in eradication continued, we increasingly modeled population immunity to transmission (Thompson, Pallansch, Duintjer Tebbens, Wassilak, & Cochi, 2013) and emphasized its importance in stopping and preventing WPV transmission, cVDPV transmission, and the evolution of OPV‐related viruses that can become cVDPVs (Duintjer Tebbens, Pallansch, Kalkowska, et al., 2013; Duintjer Tebbens,Pallansch, Kim, et al., 2013; Duintjer Tebbens, Pallansch, Wassilak, Cochi, & Thompson, 2015b; Kalkowska, Duintjer Tebbens, & Thompson, 2014a, 2014b; Thompson, 2012, 2014; Thompson & Duintjer Tebbens, 2015a; Thompson, Kalkowska, & Duintjer Tebbens, 2015). We also recommended that countries identify unvaccinated and undervaccinated subpopulations and intensify efforts to increase OPV coverage in these subpopulations, because they can sustain LPV transmission (Duintjer Tebbens et al., 2014; Duintjer Tebbens et al., 2015b; Duintjer Tebbens, Pallansch, et al., 2019; Duintjer Tebbens, Pallansch, Kalkowska, et al., 2013; Kalkowska et al., 2014a, 2014b; Kalkowska, Duintjer Tebbens, Grotto, et al., 2015; Thompson & Duintjer Tebbens, 2017c). In support of these efforts, we recommended that the GPEI ensure sufficient OPV supply and budgets to support OPV SIAs for the polio endgame (Duintjer Tebbens & Thompson, 2015, 2017d).

### 4.3 OPV Cessation

Based on full characterization of OPV‐related risks (Duintjer Tebbens et al., 2006), we recommended that the GPEI and OPV‐using countries globally‐coordinate the cessation of all use of OPV following the certification of successful WPV eradication (Duintjer Tebbens et al., 2008; Thompson & Duintjer Tebbens, 2007; Thompson et al., 2008). Following the GPEI shift to using mOPVs and bOPV in SIAs in some high‐risk countries, and our observation of the shift of significant increases in cVDPV2s (Duintjer Tebbens et al., 2013), we recommended and identified prerequisites for OPV2 cessation (Thompson & Duintjer Tebbens, 2012). Prior to OPV2 cessation, we recommended that the GPEI and countries recognize existing immunity gaps for serotype 2 and that they intensify immunization with tOPV prior to OPV2 cessation to prevent cVDPV2s before and after OPV2 cessation (Thompson & Duintjer Tebbens, 2014a). We also explored different vaccine and timing options for OPV cessation prior to OPV2 cessation, and we recommended consideration of delaying IPV introduction and delaying OPV2 cessation by a year to instead coordinate simultaneous cessation of OPV for serotypes 2 and 3 (Thompson & Duintjer Tebbens, 2015b). We demonstrated the relatively small role that IPV introduction shortly before OPV2 cessation might play with respect to mitigating the risks of cVDPV2s after OPV2 cessation, and recommended that the GPEI should not give countries a false sense of security from the introduction of IPV (Duintjer Tebbens & Thompson, 2014). We characterized the vulnerability of countries to become susceptible to reestablished transmission of an imported WPV or cVDPV or an imported Sabin OPV as a function of time since OPV cessation, and recommended coordinated OPV cessation and activities to monitor and manage OPV cessation risks (Duintjer Tebbens, Hampton, & Thompson, 2016a, 2016b, 2018). We recommended that countries should maintain high‐quality OPV SIAs using all of the remaining serotypes prior to complete OPV cessation to minimize cVDPV risks before and after complete OPV cessation (Duintjer Tebbens, Hampton, & Thompson, 2018; Duintjer Tebbens et al., 2016; Thompson & Duintjer Tebbens, 2015a). Following OPV2 cessation, we observed that the die out of serotype 2 LPVs generally behaved as our modeling expected and that tOPV intensification efforts worked in most areas (Thompson & Duintjer Tebbens, 2017b). We also identified risks and issues, which led us to recommend preparedness in the event of the potential need to restart OPV (Duintjer Tebbens & Thompson, 2018; Thompson & Duintjer Tebbens, 2017a, 2017c). Recently, we demonstrated the implications of continued LPV2 transmission as of the end of 2019, which suggested higher risks of OPV2 restart (Kalkowska, Wassilak, Cochi, Pallansch, & Thompson, 2020).

### 4.4 Outbreak Response

In the early 2000s, we observed issues associated with outbreak response timing and quality, which led us to recommend that the GPEI and countries increase the speed of detection of outbreaks, start oSIAs upon detection, and make improvements in oSIA quality (i.e., coverage and number of rounds) (Thompson et al., 2006). We repeated these themes in multiple subsequent analyses, and also recommended that the GPEI should ensure the availability of OPV for oSIAs (Duintjer Tebbens, Pallansch, Alexander, & Thompson, 2010; Duintjer Tebbens et al., 2016; Duintjer Tebbens & Thompson, 2015; Kalkowska, Duintjer Tebbens, Grotto, et al., 2015; Thompson & Duintjer Tebbens, 2008a, 2017a). We recommended that the GPEI and countries intensify serotype 2 population immunity to transmission prior to OPV2 cessation enough to prevent all cVDPV2s after OPV2 cessation (Thompson & Duintjer Tebbens, 2014a), and we assumed that they would do so in our prospective endgame modeling (Duintjer Tebbens et al., 2015a). We also recommended (and assumed in our pre‐OPV2 cessation modeling) that upon detection of transmission of VDPVs after OPV2 cessation, countries and the GPEI would view these outbreaks as emergencies and perform aggressive oSIAs (i.e., rapid, high‐quality, large scope) with mOPV2 (or tOPV if available) to quickly raise population immunity to transmission, shut down any VDPV2 outbreaks after OPV2 cessation, and decrease the probability of needing to restart OPV2 (Duintjer Tebbens et al., 2016). We recommended using tOPV if available (instead of mOPV2), because we recognized that populations susceptible to serotype 2 outbreaks also probably would be vulnerable to serotypes 1 and 3, and using tOPV would increase population immunity to all three serotypes without impacting expected die out of the serotype 2 outbreak virus based on considerations of population immunity for oSIAs involving multiple rounds (in contrast to individual immunity considerations) (Duintjer Tebbens et al., 2016). We recommended that the GPEI and countries should **not** use IPV for outbreak response, except perhaps in the context of a ring of IPV use around the population with OPV oSIAs (Duintjer Tebbens et al., 2016). We further demonstrated that using IPV is not cost‐effective when used in addition to OPV in the outbreak population after OPV cessation (i.e., IPV supplemental use provides marginal, if any, benefits and comes at high cost when added to OPV, and IPV use for oSIAs alone should not occur if OPV is available because OPV performs better for oSIAs than IPV) (Duintjer Tebbens & Thompson, 2017b). We recognized that after successful OPV cessation and exhaustion or expiration of all supplies of OPV, IPV would represent the only option for outbreak response, and consequently we used IPV for outbreak response after it represented the only option (Duintjer Tebbens et al., 2015a; Thompson & Duintjer Tebbens, 2017b), and we recommended that the GPEI and countries that self‐produce poliovirus vaccine explore options for long‐term creation of an IPV stockpile (Duintjer Tebbens et al., 2016; Duintjer Tebbens & Thompson, 2017d).

### 4.5 IPV Use

Consistent with our recommendation that the GPEI and OPV‐using countries stop all use of OPV following successful WPV eradication (Duintjer Tebbens et al., 2008; Thompson & Duintjer Tebbens, 2007; Thompson et al., 2008), we recommended investments to develop affordable and available IPV options for relatively low‐income countries if they wanted to use IPV in the polio endgame (Thompson et al., 2008). Notably, in our recommended prerequisites for OPV2 cessation, we did not include introduction of IPV as a prerequisite, because we did not expect IPV to help with the prevention of cVDPVs (Thompson & Duintjer Tebbens, 2012). We also noted that IPV use might “imply the need to look longer for potential cVDPVs, because by removing some (but not all) susceptibles and not necessarily stopping transmission, it may take longer for a case to occur that would reveal ongoing circulation of a VDPV” (Thompson & Duintjer Tebbens, 2012). Our transmission modeling has long‐recognized and explicitly included the potential for reinfection and participation in transmission of individuals with prior immunity and the small boosting effect of IPV when given to individuals previously immunized with OPV. However, our focus on modeling transmission in populations leads us to make recommendations that focus on stopping transmission, which is driven by the individuals missed by immunization, not those that we can reach, and which depends on achieving high coverage with OPV in OPV‐using countries independent of IPV use. We recognize that countries do and will make their own decisions about which vaccines they use in their national immunization programs, particularly if they need to self‐finance, and we recommended that countries carefully consider the risks, costs, and benefits of IPV for their national interests (Thompson & Duintjer Tebbens, 2014b). Although we did not recommend required use of IPV by all countries before or after OPV2 cessation or that the GPEI pays for IPV, following the adoption of the GPEI‐led, WHO global recommendation for all countries to include one dose of IPV in their RI schedules (World Health Organization, 2014), we included this strategy and its associated costs as part of the GPEI plan in our polio endgame modeling (Duintjer Tebbens et al., 2015a; Thompson & Duintjer Tebbens, 2015b). We continue to update our estimates of IPV costs and to recommend more realistic assessments of IPV costs and benefits (Duintjer Tebbens et al., 2015a; Duintjer Tebbens, Sangrujee, & Thompson, 2006; Duintjer Tebbens & Thompson, 2016b; Thompson & Duintjer Tebbens, 2014b; Thompson et al., 2008). We note that the GPEI subsequently led efforts to change the recommended polio immunization schedule globally to include a minimum of two IPV doses in all countries for a minimum of 10 years after the last OPV cessation (World Health Organization, 2017a), which implies that our future analyses will need to include this strategy as the GPEI plan, although we also did **not** recommend use of this strategy. The IPV recommendations currently do not consider cost‐effectiveness or the implications of promoting IPV on public receptivity to OPV. We actively cautioned the GPEI against any messaging that would make OPV difficult to use in countries that still need it (e.g., by introducing IPV in OPV‐using countries with the theme that IPV is a new and thus “better vaccine”). For stopping transmission in populations, OPV remains the superior vaccine.

### 4.6 Planning and Creating Polio Endgame Risk Management Options

We recognized the real threat of financial risks and the need for the GPEI budget to include enough funds to achieve its objectives, and we recommended that the GPEI partners manage polio eradication as a major project in need of stable financing (Thompson & Duintjer Tebbens, 2008b). We advised the GPEI partners not to waver on their commitment so long as polio eradication remained feasible and they remained committed (Duintjer Tebbens & Thompson, 2009; Thompson & Duintjer Tebbens, 2007), and we performed analyses that helped to make the case for investments (Duintjer Tebbens et al., 2011; Duintjer Tebbens et al., 2015a). In our analysis of the polio endgame for 2013–2052, we focused on identifying and we recommended risk management strategies that offered a very high probability for a successful polio endgame and we assumed that the GPEI and countries would implement all of our recommendations in our analyses (Duintjer Tebbens et al., 2015a). Notably, our analyses characterized risks as a function of GPEI actions, not as absolute estimates, and we emphasized that models could not predict with certainty where or when events would occur, because the future depends on the choices that countries and GPEI leaders make. In our analysis that explored the economics of temporary recommendations for international travelers from potential polio exporting countries, we recommended expanding the application of the international travel recommendations to include countries with demonstrated WPV1 circulation instead of waiting for documented WPV1 exportations (Duintjer Tebbens & Thompson, 2017c). We recommended investments to support the development of polio antiviral drugs (PAVDs) and screening for iVDPV excreters to create an option to potentially manage iVDPV risks (Duintjer Tebbens, Pallansch, & Thompson, 2015; Duintjer Tebbens & Thompson, 2017a). We also recommended investment in research and development of a new polio vaccine that would function like OPV but with reduced risks, which we modeled with ideal attributes (Duintjer Tebbens & Thompson, 2016a). We identified the potential need to restart OPV after OPV cessation (Duintjer Tebbens et al., 2015a), characterized the risks that could lead to OPV restart (Duintjer Tebbens & Thompson, 2018), and recommended that the GPEI develop contingency plans for OPV restart (Duintjer Tebbens & Thompson, 2018; Thompson & Kalkowska, 2019). Finally, we recommended investment and attention to risk management activities to manage containment risks (Duintjer Tebbens, Kalkowska, & Thompson, 2018; Duintjer Tebbens et al., 2006; Duintjer Tebbens et al., 2015a).

## 5 OUR RECOMMENDATIONS COMPARED TO THE GPEI PATH AND ACTUAL EXPERIENCE

The GPEI and countries adopted and implemented many of our recommendations (e.g., improving the timeliness of oSIAs in the mid‐2000s, globally coordinating OPV cessation, investing in the development of some risk management options, including the development of vaccine stockpiles, PAVDs, lower‐cost IPV, new strains of OPV, etc.), and implementation of international travel immunization recommendations (Thompson, 2013; Thompson, Duintjer Tebbens, Pallansch, Wassilak, & Cochi, 2015). However, instead of focusing on failure to vaccinate and OPV SIA performance, since the mid‐2000s, the GPEI has focused considerable attention on vaccine failure, and invested considerable resources in developing tailored vaccines (e.g., first mOPV then bOPV then IPV). Based on our modeling, these diversions did not accelerate eradication (Duintjer Tebbens, Pallansch, et al., 2019; Kalkowska et al., 2014a, 2014b) and the vaccines used performed worse overall than tOPV, because they created immunity gaps that led to later problems (e.g., using mOPV1 allowed WPV3 outbreaks to occur, and using of mOPV1, mOPV3, and bOPV opened up immunity gaps to serotype 2 in areas with low coverage, which led to cVDPV2s, and using IPV does not stimulate the same type of immunity as OPV). These problems occurred without significantly improving population immunity to transmission for serotype 1 (Duintjer Tebbens et al., 2013; Duintjer Tebbens et al., 2015b; Duintjer Tebbens & Thompson, 2017d; Kalkowska et al., 2014a, 2014b; Thompson & Duintjer Tebbens, 2017c). Notably, as of early 2020, WPV1 continues to circulate. In addition, although our modeling suggested that the GPEI should invest its resources in working with countries to achieve and maintain high population immunity to transmission with tOPV prior to OPV2 cessation (Thompson & Duintjer Tebbens, 2014a), which occurred and worked in most countries, not all countries achieved sufficiently high population immunity to transmission for serotype 2 prior to OPV2 cessation to prevent all cVDPV2s (Duintjer Tebbens & Thompson, 2018). Notably, Pakistan and Afghanistan used fewer tOPV rounds than recommended in the run up to OPV2 cessation (Duintjer Tebbens, Pallansch, et al., 2019; Duintjer Tebbens & Thompson, 2019), and they and the GPEI invested significantly in introducing expensive IPV in their national immunization programs. The use of IPV in Pakistan and Afghanistan included some use in SIAs, which our modeling suggested was not an effective strategy or a cost‐effective use of resources and was insufficient to prevent a cVDPV2 or to accelerate WPV1 elimination (Duintjer Tebbens, Pallansch, et al., 2019).

The discovery of transmission of WPV1 in northeast Nigeria in late 2016, despite apparent indicators of sufficient AFP surveillance quality suggesting no transmission prior to that time, indicated degradation of surveillance quality. These events in part appear to reflect actions by the GPEI partners to begin implementing post‐eradication transition plans prior to actual eradication in Nigeria at the same time of significant population and programmatic disruptions occurred due to insecurity. While the GPEI invested in some expansion of environmental surveillance in some areas with unknown quality, some degradation of the quality of AFP surveillance has occurred. Recognizing our optimism about sustained high‐quality polio surveillance information for the polio endgame, our future modeling will seek to account for actual GPEI and country plans for surveillance, and will explicitly characterize poor‐quality (or no) environmental surveillance in inaccessible un‐ and undervaccinated subpopulations (Kalkowska et al., 2020).

With respect to post OPV2 cessation outbreak response, the GPEI and countries did not respond to signals of serotype 2 LPV transmission after OPV2 cessation as emergencies as we expected and recommended. They also did not use tOPV, even though it was available shortly after OPV2 cessation, in part, because Pakistan and Afghanistan did not perform as many tOPV SIAs prior to OPV2 cessation as we expected or recommended. In theory, the available tOPV could have been used in Pakistan and Afghanistan shortly before or after its OPV2 cessation. In practice, the GPEI and countries found it unacceptable to use tOPV after OPV2 cessation. Part of this reluctance came from the demonizing of tOPV by the GPEI and countries in the run up to OPV2 cessation to encourage the rapid removal of tOPV from supply chains after OPV2 cessation. In addition, the GPEI and countries have demonstrated notable reluctance to use mOPV2, despite plans and preparations prior to OPV2 cessation for its use for outbreak response. While the GPEI and countries performed some oSIAs with mOPV2 aggressively, in some cases, they performed late, low‐quality, and small‐scope SIAs with mOPV2. In addition, instead of multiple oSIA rounds followed by again stopping mOPV2 use, the GPEI and countries continue to introduce mOPV2 as of the time of writing in small‐ and low‐quality oSIAs (e.g., trickling it in). In addition, research that showed high seroconversion to mOPV2 by vaccine recipients with two doses (i.e., good induction of individual immunity) (Zaman et al., 2018), led the GPEI and countries to think that they could perform fewer oSIA rounds independent of the quality of the rounds than we recommended in our modeling. Our modeling assumed high seroconversion to mOPV2 by individuals who received it (i.e., consistent with the evidence on immunogenicity of mOPV in individual vaccine recipients), but recommended more aggressive outbreak response with more rounds due to our focus on increasing population immunity to transmission to stop the outbreak and prevent future cVDPVs, which depends on achieving high coverage and timeliness (i.e., quality) and sufficient scope (scale and frequency) to stop transmission. We also assumed that oSIAs would occur and then mOPV2 use would stop to allow die out again to support OPV2 cessation, not that mOPV2 use would continue. Our future modeling will seek to account for more realistic oSIA performance, and will explicitly characterize poor quality and smaller scope efforts, which will change cVDPV risks in the polio endgame (Kalkowska et al., 2020).

We also recognize that in the absence of budgeted oSIA funds, the GPEI may need to use funds originally budgeted for pSIAs to support oSIAs. This may impact the conduct of pSIAs, and thus the maintenance of population immunity to transmission with OPV and vulnerability to future outbreaks. Finally, in countries that need OPV SIAs to maintain high population immunity to transmission, misperceptions that the introduction of IPV in RI provides a good substitute for continued OPV SIAs may also impact OPV SIA conduct and quality.

Our modeling of the polio endgame anticipated lower risks of cVDPVs and higher risks of iVDPVs than observed to date (Duintjer Tebbens et al., 2015a), in large part because we assumed that countries and the GPEI would conduct enough tOPV SIAs prior to OPV2 cessation to prevent cVDPV2s and respond very aggressively to shut down any transmission that emerged after OPV2 cessation. Now four years since OPV2 cessation, countries and the GPEI did not take these actions uniformly.

Significant uncertainty remains about the transmissibility and paralytic potential of iVDPV viruses, and our modeling conservatively assumes that iVDPVs can behave like cVDPVs. However, using a less conservative assumption (e.g., assuming iVDPV virus introduction into populations occur with less‐than‐fully reverted viruses) significantly affects our modeling results (Duintjer Tebbens & Thompson, 2017a). In the context of modeling disappearing cVDPV2 risks, iVDPV risks emerged as a concern in our prior overall global risk modeling. To date, the empirical evidence of reintroduction of transmission of LPVs by an iVDPV excreter remains highly limited, and this supported us using a less conservative assumption about the nature of iVDPV introductions in our updated modeling (Kalkowska et al., 2020).

The continued transmission of WPV1 in Pakistan and Afghanistan to date clearly indicates a failure to develop strategies to achieve and maintain high population immunity to transmission. We recommended proactive, aggressive OPV pSIAs, but the GPEI and these countries appear to have focused much more on introducing IPV, which our modeling suggests is not effective in stopping transmission in countries that the GPEI supports. In addition, in recent years, the GPEI efforts to pull back on investments in pSIAs and surveillance in some countries, with the assumptions that countries will pick up the costs for sustaining the activities, has led and may lead to reduced program capacity and quality in some areas, which implies significantly more risk than we previously modeled. In addition, if countries do not plan for and conduct pSIAs, the lack of planned vaccine demand may lead to insufficient OPV production and shortages of OPV during the remainder of the polio endgame. Without sufficient OPV, the GPEI will not achieve polio eradication.

At the beginning of 2019, upon recognition that it would not achieve the objectives of the 2013–2019 GPEI Strategic Plan, the GPEI released a new Strategic Plan for 2019–2023. However, from the perspective of modelers, that plan does not provide clear guidance about how the GPEI and countries with continued WPV and cVDPV transmission will end their transmission and prevent future transmission or provide a strategy for how the GPEI and countries will handle ongoing serotype 2 LPV transmission.

The GPEI also handled IPV very differently from what we recommended. The GPEI led efforts to make at least one dose of IPV a recommended vaccine in every national immunization program as a prerequisite to globally‐coordinated OPV2 cessation (World Health Organization, 2014), and took on the responsibility for financially supporting IPV introduction into GPEI‐supported countries. The GPEI prioritized introduction of IPV into countries at high risk of poliovirus transmission (i.e., the endemic and recently endemic countries), which effectively allocated IPV doses to the worst performing health systems (i.e., the countries least prepared to use it and least likely to benefit from its use), at the expense of taking IPV away from countries with higher performing health systems that face greater risks associated with iVDPV excreters. This approach may also have distorted the market, by making IPV unavailable to countries in a better position to pay for it, which would probably have supported the development of more typical tiered‐pricing and provided better motivation for manufacturers to increase IPV production capacity. Despite these issues, IPV supply shortages, and continued high IPV prices, the GPEI led efforts to change the recommended polio immunization schedule globally to include a minimum of two IPV doses in all countries for a minimum of 10 years after the last OPV cessation (World Health Organization, 2017a). This recommendation occurred without the benefit of any analyses exploring the impact of IPV use on risks, transmission reduction benefits, or vaccine supplies or costs, and with no timeline for implementation. If the GPEI applies the requirement of two doses of IPV as a prerequisite to complete OPV cessation or prior to globally‐coordinating OPV3 cessation, then this could effectively make the GPEI a very expensive control program instead of an eradication initiative, independent of whether or when the GPEI stops WPV1 and/or cVDPV2s. The transition to the recommended use of a minimum of now one IPV dose (and at some point, two IPV doses) significantly increased the costs of the GPEI by making the baseline comparator one IPV dose plus whatever OPV doses are needed, instead of just the OPV doses needed.

## 6 LESSONS FROM REFLECTIONS ON OUR MODELING OF THE POLIO ENDGAME FOUR YEARS AFTER OPV2 CESSATION

We summarize key lessons learned from this exercise for our future modeling as follows (and indicate whether we implemented this in our recent model update [Kalkowska et al., 2020]):

1. Focus on prospectively modeling actual practice and plans, and do not assume that the GPEI or countries will behave optimally and/or benefit from good luck (implemented by using current estimates for immunization and surveillance, including oSIA inputs that achieve lower expected coverage and reduced scope as occurred after OPV2 cessation).
2. Recognize the higher risks of reintroduction of OPV after its cessation than observed in rare instances prior to OPV cessation (partially implemented by increasing the risks associated with reintroduction of vaccine not removed from the supply chain, but probably not increased enough with respect to intentional contamination of the supply chain given actual events, which include release of vaccine contaminated by OPV2 in India [PTI, 2018; Thacker, 2018] and atypical and unexpected introduction of OPV2 in vaccine in parts of Africa and Pakistan leading to the cases observed in late 2019) (Macklin et al., 2020).
3. Account for limited supplies of vaccines (not implemented yet, our recent model update focuses on running the model with unlimited supplies to assess implied vaccine needs, future work will need to consider the implications of actual supply limitations).
4. Account for the use of different vaccines (implemented for all currently licensed vaccines, not implemented for potential new vaccines, including new or novel strains of OPV [nOPV] under development [Van Damme et al., 2019]).
5. Recognize the significant costs of IPV for the GPEI and OPV‐using countries (partially implemented, updated model includes the introduction of one dose of IPV that occurred in OPV‐using countries starting in 2015, but does not include use of a minimum RI schedule with more IPV doses in OPV‐using countries).
6. Account for likely degradation of AFP surveillance and introduction of environmental surveillance (ES, implemented, although uncertainty remains about future support and the use of ES to trigger outbreak response).
7. Anticipate that countries can become unstable and thus weak links for polio eradication (e.g., displacement of individuals from conflict and/or events) (partially implemented by modeling un‐ and undervaccinated populations in countries currently recognized as or anticipated to be weak links, but may not include all countries that will emerge as weak links for the polio endgame).
8. Appreciate that the risks of reintroduction from an iVDPV excreter may imply introduction of the LPV as a less‐than fully transmissible VDPV (implemented by assuming iVDPV introductions occur with the transmissibility and neurovirulence equivalent to OPV evolution state 10, although uncertainty remains).

We summarize key programmatic lessons learned from OPV2 cessation as follows:

1. All countries that use OPV should use only the formulation that includes all of the serotypes contemporaneously allowed (i.e., use bOPV after OPV2 cessation, but not mOPV1 since this opens up an immunity gap for serotype 3 population immunity).
2. OPV cessation should be coordinated.
3. Countries that perform OPV SIAs should continue to maintain them until the time of coordinated OPV cessation to keep population immunity to transmission high and to maintain capacity for OPV oSIAs. As needed, and particularly if maintenance of OPV pSIAs does not occur, countries should conduct intensified OPV pSIAs prior to globally coordinated cessation of any OPV serotype(s) to ensure high population immunity to transmission prior to homotypic OPV cessation.
4. All OPV containing the serotype stopped should be aggressively and completely removed from the supply chain within six months of the global OPV cessation date.
5. Surveillance for transmission of any circulating LPVs after OPV cessation must remain high.
6. Response to any VDPVs detected after the homotypic LPVs should have died out should recognize the emergency nature of the outbreak and occur quickly, with sufficient size, number of rounds, and coverage achieved to rapidly stop all transmission.
7. After conducting OPV oSIAs to stop a post cessation outbreak, all OPV supplied for use for the oSIAs that remains unused should be completely withdrawn from the supply chain.
8. IPV should not be a prerequisite for OPV‐using countries as part of polio eradication. IPV should remain an option for those countries that wish to use it, but IPV should not be considered as a tool that would help to reduce LPV transmission in the countries that need any OPV.
9. Develop communication tools and strategies that will ensure the rapid use of an OPV serotype after its cessation in the event of detection of an outbreak after cessation.
10. Ensure sufficient resources (i.e., financial, human, and vaccine supply) to conduct all cost‐effective activities, and do not waste resources on interventions with little expected impact.

## 7 DISCUSSION

Modelers support decisionmakers, but do not make the decisions. As demonstrated, our modeling results only influenced GPEI and national decisions in some cases, with other factors or the results from other modelers carrying more weight in others. Thus, while we focused primarily on our modeling, we recognize that the GPEI engaged multiple modeling groups that use(d) different approaches and sometimes provide(d) conflicting insights or recommendations. In some instances, in which our recommendations differed from the path the GPEI took, other modeling groups made recommendations that supported the different path. We also observed that decisionmakers can selectively use the results of different models to support the policies that they prefer, and point to limitations of the models as the reasons for not using the model results that they do not prefer. While the GPEI may find some advantage of multiple modeling groups when the recommendations of the independent groups agree, the lack of agreement may compel the GPEI to choose among competing recommendations when they do not agree.

Disagreements from the polio modeling groups can reflect different premises and/or modeling approaches. In addition, the limitations of models imply that they can produce recommendations that will later prove invalid. Validation exercises help us as modelers to take stock of where we got things right and wrong and help us to learn.

One of the most significant lessons from this analysis for us comes in the form of ensuring that our models focus on the actual plans of action and realistic expectations of performance, instead of (or in addition to) identification of what we see as optimal strategies. We also recognize that we cannot assume that decisionmakers will accept and/or can implement our recommendations. In this context, one of the key implications of attempting to model current activities and plans leads to our updated global model, which shows poor performance in the future and a failure to eradicate WPV1, control cVDPV2s, and avoid the need to restart production of OPV2 (Kalkowska et al., 2020). Given the current path of the GPEI, we recommend that the GPEI partners and countries consider whether long‐term control with OPV or IPV will represent the preferred strategy for relatively lower income countries in the context of realistic assumptions about coverage, costs, and willingness‐to‐pay for vaccine, and that the GPEI and countries prepare for OPV2 restart. The GPEI currently places high hopes on the introduction of nOPV, which developers engineered to make less likely to revert to the neurovirulent genotype than Sabin OPV2 (Van Damme et al., 2019). However, significant uncertainty remains about the regulatory and development path of new vaccines with novel OPV strains and how they will perform when delivered to real populations if and when used.

Modeling suggests that the GPEI can still achieve eradication of all WPVs (Kalkowska & Thompson, 2020). However, if the GPEI and countries cannot achieve and maintain high population immunity to transmission for all three serotypes until successfully stopping all OPV and ensure that they successfully stop all OPV, then they may need to declare WPV1 eradication as not feasible and/or abandon the strategy of OPV cessation after WPV eradication altogether. This would represent a substantial disappointment and missed global opportunity, not only for polio, but probably for future global public health major projects, including other disease eradication efforts. We recently suggested that WPV1 eradication is possible, with a commitment to high coverage bOPV use in Pakistan and Afghanistan (Kalkowska & Thompson, 2020). With the expenses of the GPEI mounting at an accelerated rate given its adoption of IPV and the failure to stop the transmission of WPV1 and cVDPV2s to date, challenges to the GPEI will likely include financial risks that may prove particularly problematic. In the face of limited financial resources, OPV use should represent a much higher priority than IPV use.

Eradication represents a global public good that offers the greatest possible equity of all individuals benefitting from the absence of LPVs independent of their immunity. Since 2015, the GPEI appears to be on a path that places the highest value on equity of access to IPV, which from a modeling perspective makes it more of a control program. Using IPV for control is much more expensive than using OPV for control, and control can occur with relatively higher or lower average immunization coverage, which will impact the relative health and financial benefits and costs (Thompson & Duintjer Tebbens, 2007).

While we optimistically believe that polio eradication remains possible and that it should be done, we also believe that the GPEI is not on track to achieve it. Modeling can identify strategies that could still be implemented to finish the job more cost‐effectively. However, the GPEI and countries will need to prioritize the use of resources on delivering OPV to increase population immunity to transmission, and maintain (and rebuild in some cases) the GPEI as a sufficiently vertical and performance‐driven effort that it produces accountable results. Experience suggests that focusing on the nebulous and undefined concept of system strengthening, which may or may not lead to measurable or accountable performance on specific OPV coverage objectives, will not get the job done quickly, if at all. As financial pressures increase, the GPEI may also need to make a successful case to donors that it has learned from its mistakes and that it will spend the additional funds required cost‐effectively to finish the job. Taking IPV out of the GPEI to make it separate from eradication and not a prerequisite for WPV eradication or OPV cessation would likely help to focus the GPEI on OPV and ending all LPV transmission. Equity would come from achieving the vision of polio eradication to end all LPV transmission. In contrast, equity will probably not come from delivering unequal coverage of expensive vaccines that work differentially in different populations. Some individuals will always remain unvaccinated, as still occurs now in countries with the best health systems, but this does not matter if eradication succeeds.

Eradication programs, like the GPEI, can play a significant role in providing lessons learned and promoting global equity for other diseases (Thompson & Duintjer Tebbens, 2011). Unfortunately, we failed to fully learn the lessons from SARS‐CoV, and despite preventing Severe Acute Respiratory Syndrome coronavirus (SARS‐CoV) from becoming epidemic in 2003, the world failed to “ensure the proper incentives for early detection, reporting, coordination, and action related to the management of emerging diseases” (Thompson & Duintjer Tebbens, 2011, p. 119) following the detection of SARS‐CoV‐2 in 2019. Widespread transmission of SARS‐CoV‐2 virus in early 2020 led to the declaration of COVID‐19 as pandemic. Future analyses will need to explore the implications of the COVID‐19 pandemic on polio eradication and other disease control and elimination efforts.
